# Prevalence of coccidian infection in rabbit farms in North Algeria

**DOI:** 10.14202/vetworld.2018.1569-1573

**Published:** 2018-11-12

**Authors:** Samia Maziz-Bettahar, Miriem Aissi, Hacina Ainbaziz, Mohamed Sadek Bachene, Safia Zenia, Fairouz Ghisani

**Affiliations:** 1 Clinical Department, Institute of Veterinary Science, University of Blida 1, Ouled Yaich 9000 Blida, Algeria; 2 Higher National Veterinary School, Laboratory Research of Health and Animal Production, BP161, Rue Issad Abbes, Oued Smar, Algiers, Algeria; 3 Department of Nature and Life Sciences, Dr. Yahia Farès University, Médéa, Algeria; 4 Renewable Energy and Environmental, Faculty of Technology, University of Blida 1, Ouled Yaich 9000 Blida, Algeria

**Keywords:** Algeria, coccidiosis, *Eimeria*, oocysts, prevalence, rabbit

## Abstract

**Aim:**

The aim of the present study was to determine the prevalence and intensity of rabbit coccidiosis (*Oryctolagus cuniculus*) in North Algeria.

**Materials and Methods:**

During the study, 40 rabbit farms were investigated. The farms are located in the provinces of Tizi Ouzou, Médéa, and Djelfa which distributed, respectively, into three regions: East Tell Atlas Mountains, Central Tell Atlas Mountains, and High Plateaus. The number of oocyst per gram of feces (OPG) was determined by McMaster technique, and the *Eimeria* species were identified using morphological criteria.

**Results:**

In the farms investigated, the prevalence of coccidian infection was estimated to 90% (80.7-99.3%) in rabbits after weaning. The classification of the farms according to their parasite load allowed us to show that 37.5% of the prospective farms have an oocyst excretion between 10^4^ and 5×10^4^ oocysts per gram and 22.5% excrete >5×10^4^ oocysts per gram. Excretion levels by region show that the region of East Tel Atlas Mountains ranks first with 79% of farms with a parasitic load >10^4^ coccidians compared to the regions of Central Tel Atlas Mountains and High Plateaus. In total, eight species of *Eimeria* were identified from oocyst-positive samples. Mixed infections with four *Eimeria* species were common. *E. magna* is the dominant species in comparison with *E. media* and *E. irresidua* with respective frequencies of 42.5% and 17.6% and 14.9% (p<0.001). Our results showed that the farms using anticoccidial drugs for their rabbits were low (25%) and the percentage of farms with poor hygienic conditions was 65%. There was a significant association between increased oocysts excretion and control measures of coccidian infection.

**Conclusion:**

The study revealed an overall prevalence of 90% in the three Algerian regions. A strong association was observed between *Eimeria* infection and hygienic status and preventional chemotherapy.

## Introduction

In Algeria, rabbit breeding is ancient, according to a traditional method, which is still present, nowadays [1]. Rational breeding which appeared in 1987 was introduced by the government, to improve the animal protein consumption of the Algerian people [2].

However, the installation of rabbit farming did not reach its goal for multiple reasons such as lack of specific sanitary conditions for rabbits as well as parasitosis, which is a permanent presence of pathology. The coccidiosis is the most common diseases in rabbits and caused by protozoa of the genus *Eimeria* which is developed in the digestive tract. Widely described in numerous publications [3-7], they are responsible for serious disturbances resulting in significant economic losses. All domestic rabbits can be infected by coccidia, but the weaned rabbits are the most sensitive [8]. In Algeria, few studies have been carried out on the pathogen. Only the study carried out by Henneb and Aissi [9] revealed the excretion of oocysts in rabbits during lactation and their offspring. The study conducted by Bachene *et al*. [10] confirmed the pathogenicity of *Eimeria magna* within the local population of rabbits.

However, to the best of our knowledge, there is no published report of prevalence of *Eimeria* infection in Algerian rabbit farms. The aim of the present study was to determine the prevalence, parasitic status, and *Eimeria* species present and control measures of coccidian infection in rabbit after weaning.

## Materials and Methods

### Ethical approval

This study was based on the fecal sample collection only; hence, the ethical approval was not required. The fecal samples were collected under the cages of the rabbits with the prior consent of the farmers.

### Study farms and rabbit populations

**Figure-1:**
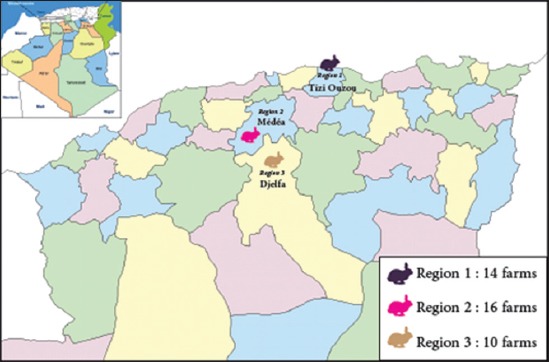
Map of North Algeria showing the region and geographical distribution of rabbit farms investigated in the study.

In the present study, 40 small farms of 25 breeding females belonging for the majority of private producers were investigated in North Algeria, where rabbit breeding has been developed. The farms belong to the provinces of, Tizi Ouzou, Médéa, and Djelfa which are part of the three following regions: Region 1 includes East Tel Atlas Mountains (Tizi Ouzou), Region 2: Central Tel Atlas Mountains (Médéa), and Region 3: Central region of the High Plateaus (Djelfa) (Figure-1). Rabbit populations were Californian or New Zealand breeds, local, hybrid, or cross-breeding. These animals were housed in a wire cage put in hangars or recovery habitats with the absence of environmental microclimatic conditions control. The cages housing the breeding females are placed in the same room. The commercial pelleted feed was given *ad libitum* which did not include anticoccidials.

### Fecal samples

A total of 273 fecal samples were collected from weaned rabbits (40-50 days of age) during the year of 2009 to 2011 between January and June These months of samples correspond to a high presence of weaned rabbits in the fattening. For each farm visited, only one sample was carried out, and individual fresh fecal samples were collected in containers set under the cages 24 h before. Then, the feces harvested have been moistened, packed in plastic bags, stored, and refrigerated at 4°C until examination. Information regarding hygienic conditions and chemoprevention were recorded.

### Parasitological analysis

For each collection and after homogenization, 300 g of sample were mixed in 1500 ml of water, and then 40 g of the mixture was put into 60 ml of saturated salt solution. The suspension was transferred with a Pasteur pipette into a McMaster counting chamber (20 columns). The oocyst per gram (OPG) was calculated to estimate the degree of infection [11]. The suspension of oocysts used for the enumeration of coccidia was filtered with a pass tea, and then the filtrate collected was subjected to three washes by sedimentation to clean the fecal suspension. The second wash, a drop of bleach diluted to 12° is added to the suspension to eliminate the bacteria. Once collected, the oocysts have been sporulated in a 2.5% potassium dichromate solution at ambient temperature of laboratory (24-26°C) using Erlenmeyer flask. A daily basis check proceeded until sporulation of the oocysts. The diagnosis of different encountered species has been carried out based on the descriptions reported by Eckert *et al*. [12].

### Statistical analysis

Data were entered using a Microsoft Excel® 2007, and statistical analysis was performed in R version 3.5.0 (the R Foundation for Statistical Computing) [13] using package Rcmdr: R Commander version 2.4-4 [14]. Measures of association were based on the Chi-squared and Fisher’s exact test, and the averages of the species were tested by analysis of variance. p<0.05 was considered as statistically significant.

## Results

### Prevalence and parasitic status

In the three Algerian regions investigated, the prevalence of rabbit coccidiosis was estimated at 90% (95% confidence interval 80.7-99.3%). The parasite was presented in 36/40 farms prospected (Table-1). When reassessed according to regions, the prevalence varied from 100% (10/10) in High Plateaus, to 92.9% (13/14) in East Tell Atlas, and to 81.3% (13/16) in Central Tell Atlas. The level of infection with coccidian OPG of faces is shown in Table-2. 60% of farms (n=40) surveyed have an oocyst excretion over 10^4^ OPG, and 22.5% excrete more than 5×10^4^ OPG. The rest of the farms (32.5%) have a lower excretion to 5×10^3^ OPG. The majority of farms of East Tell Atlas excrete >5×10^3^ OPG with a peak of the order of 10^4^ to <5×10^4^ OPG. The farms of Central Tell Atlas are characterized by 18.8% without coccidia and a peak of OPG in the order of 10^4^-<5×10^4^. 30% of the farms in High Plateaus excrete >5×10^4^ OPG, and 40% are <5×10^3^ OPG. The intensity of infection in East Tell Atlas was significantly (p<0.05) higher than in Central Tell Atlas and High Plateaus.

**Table-1:** Regional prevalence of coccidian infection in Algerian rabbit farms.

Region	Province	Farms x/n	Percentage
East Tell Atlas	Tizi Ouzou	13/14	92.9
Central Tell Atlas	Médéa	13/16	81.3
High Plateaus	Djelfa	10/10	100
Total		36/40	90.0

**Table-2:** The percentage distribution of the farms in three regions of Algeria according to the intensity of coccidian infection classes.

OPG class	Region and number of farms examined			

	East Tell Atlas n=14	Central Tell Atlas n=16	High Plateaus n=10	All regions n=40
0-<10^2^	7.1	18.8	0.0	10.0
10^2^-<5×10^3^	0.0	31.2	40.0	22.5
5×10^3^-<10^4^	14.3	0.0	10.0	7.5
10^4^-<5×10^4^	50.0	37.5	20.0	37.5
>5×10^4^	28.6	12.5	30.0	22.5

OPG=Oocysts per gram

### Prevalence of Eimeria species

**Figure-2:**
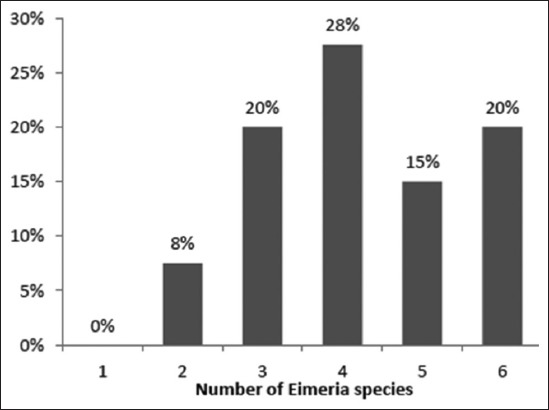
Percentage of mixed infections with different *Eimeria* species in Algerian rabbit farms.

The study disclosed the presence of eight species of *Eimeria*, namely *E. magna*, E. *media*, *E. irresidua*, *E*. *perforans*, *E. stiedai*, *E*. *coecicola*, *E. intestinalis*, and *E. piriformis*. *E. magna* is the dominant species before *E. media* and *E. irresidua* with respective frequencies of 42.5% and 17.6-14.9% (p<0.001). The weakly species encountered are *E. perforans* (7.8%), *E. stiedai* (4.1%), *E*. *coecicola* (1.7%), *E. intestinalis* (0.9%), and *E. piriformis* (0.6%). Mixed infection with two to six species of *Eimeria* occurred most frequently, and 63% of specimens contained four to six species (Figure-2). In East Tell Atlas, *E*. *intestinalis* and *E. piriformis* were not detected. *E. magna* was the most prevalent (30.5%), followed, respectively, by *E. irresidua* (20.4%), *E. media* (19.8%), and *E. stiedai* (10.8%). In Central Tell Atlas, all eight species were detected, and *E. magna* was the dominant species (41.1%), followed, respectively, by *E. irresidua* (14.6%), *E. media* (13.9%), and *E. perforans* (7%). In High Plateaus, there was no finding *E. coecicola*, *E. intestinalis*, and *E. piriformis*. *E. magna* was the most prevalent (61.7%) species, followed, respectively, by *E. media* (20.3%), *E. perforans* (9.6%), and *E. irresidua* (7.6%) (Table-3).

**Table-3:** Prevalence of *Eimeria* species in three regions of Algeria.

Species	East Tell Atlas	Central Tell Atlas	High Plateaus
*E. magna*	30.5±4.8	41.1±10.4	61.7±7.4
*E. media*	19.8±3.2	13.9±4.9	20.3±4.6
*E. irresidua*	20.4±4.0	14.6±6.2	7.6±2.5
*E. perforans*	7.4±1.7	7.0±3.6	9.6±1.9
*E. stiedai*	10.8±4.6	0.4±0.3	0.5±0.5
*E. coecicola*	4.1±1.4	0.8±0.4	0.0
*E. intestinalis*	0.0	2.1±1.2	0.0
*E. piriformis*	0.0	1.4±0.7	0.0

E. magna=Eimeria magna, E. media=Eimeria media, E. irresidua=Eimeria irresidua, E. perforans=Eimeria perforans, E. stiedai=Eimeria stiedai, E. coecicola=Eimeria coecicola, E. intestinalis=Eimeria intestinalis, E. piriformis=Eimeria piriformis

### Control measures of coccidian infection in rabbit farms

The evaluation of hygienic conditions and the use of anticoccidial drugs on the intensity of coccidian infection were recorded in Table-4. The results showed a strong association between hygienic conditions and increased oocysts excretion. The percentage of farms with poor hygienic conditions was 65%, and the majority had OPG >5×10^3^. The farms using anticoccidial drugs for their rabbits were low (25%), and there was a significant association between increased oocysts excretion and no anticoccidial drugs usage.

**Table-4:** Risk of intensity of coccidian infection in Algerian rabbit farms.

Risk factors	Oocysts excretion per gram OPG>5×10^3^	Odds ratio	95% confidence interval	p-value
Hygiene				<0.0001
Good	3/14 (21.4)	1		
Poor	24/26 (92.3)	37.1	5.1-506.3	
Anticoccidial drugs				0.0006
Yes	2/10 (20.0)	1		
No	25/30 (83.3)	17.9	2.6-222.5	

OPG=Oocysts per gram

## Discussion

Coccidiosis constitutes the major etiology of intestinal disorders in the rabbit that affects mainly young rabbits after weaning [15,16]. The present study disclosed a high prevalence of coccidian infection in young rabbit after weaning from three regions of Algeria. The high prevalence may be explained by the role mothers play in transmitting the infection to their litters [9,17], and the young rabbits after weaning are lower resistance and less immunity to coccidian infection than in older animals[8].

The classification of farms according to their parasitic status has allowed us to identify farms that are in a pathological situation [18] so that more than half of farms record oocyst excretions from 10^4^ to >5×10^4^ OPG. The East Tell Atlas region ranks first with 79% of farms counting >10^4^ coccidia compared to the Central Tell Atlas region where 18.8% of farms have no coccidia, and 40% of High Plateaus farms are below 5×10^3^ OPG. Our results showed that control of rabbit coccidiosis is entirely dependent on chemotherapy and hygienic conditions of farms (Table-4). The efficacy of anticoccidial drugs has been confirmed in various studies [19-22], mixed in feeding pellets or drinking water. The administration of anticoccidial drugs in drinking water was observed in 25% of farms surveyed, mostly using sulfonamides which contributed to reducing the level of infection. However, 5% of farms excretion levels are high; the reason is probably due to the use of the anticoccidial drug when clinical signs of coccidiosis appeared, and the treatment is usually not very successful [21].

Moreover, in rabbit breeding, all therapy should concern not only the young growing rabbits but also the nursing females because it is essential during the week preceding weaning that the contamination from mother to young rabbits takes place [23].

An alternative approach to control coccidian infection is hygienic measures. Indeed, the majority of rabbit farms where hygienic conditions were poor had high levels of excretion. Gonzalez-Redondo *et al*. [24] confirmed that a fair control of hygienic conditions is sufficient to maintain a low level of coccidian and Schlolaut *et al*. [25] indicated that housing conditions could have an impact on health of rabbits. Multiple infections were common during our study, 90% of infected animals carried, two to six species of *Eimeria*. The natural infections with a single *Eimeria* species are rare [26,27].

On the 11 species of coccidia described in the rabbit [12,28-30], eight species have been identified. *E. magna* is the dominant species before *E. media* and *E. irresidua*. These three species are pathogenic for the rabbit. They are responsible for the depression of growth as well as the possibility of the occurrence to clinical coccidiosis [4,5,11,31]. During our study, 28% of the farmers declared the observation of diarrhea in their rabbits. Our results revealed high OPG values in weaned rabbits which would explain to clinical coccidiosis. However, the occurrence of diarrhea may also have a bacterial origin [32].

## Conclusion

Through our study, we have highlighted the presence of coccidia in 36 farms on a total of 40. The intensity of infection was divided into different ways. We have noted that more than half of the farms have oocyst excretions of >5×10^3^ oocysts per gram. Eight species of coccidia were identified, with a predominance of *E. magna*. Preventive measures such as the prophylactic use of anticoccidial drugs and hygienic conditions have been determining the factors on the control of rabbit coccidiosis. Future studies undergoing epidemiological study of rabbit coccidiosis such as the influence of age, breeds, and season will have to be undertaken.

## Authors’ Contributions

SM conducted the study, drafted, and revised the manuscript. MA and HA designed and supervised the work. SM and SZ analyzed the data. MSB provided support assistance to the study. FG revised the manuscript. All authors read and approved the final manuscript.
